# Moderate performance of serum S100A12, in distinguishing inflammatory bowel disease from irritable bowel syndrome

**DOI:** 10.1186/1471-230X-10-118

**Published:** 2010-10-14

**Authors:** Anastassios C Manolakis, Andreas N Kapsoritakis, Panagiotis Georgoulias, Chara Tzavara, Varvara Valotassiou, Anastasia Kapsoritaki, Spyros P Potamianos

**Affiliations:** 1 Department of Gastroenterology, University of Thessaly, School of Medicine, Larissa, 41110, Greece; 2 Laboratory of Nuclear Medicine, University of Thessaly, School of Medicine, Larissa, 41110, Greece; 3 Center for Health Services Research, University of Athens, School of Medicine, Athens, 11527, Greece

## Abstract

**Background:**

S100A12, a calcium-binding proinflammatory protein secreted by granulocytes, has been associated with different diseases of inflammatory origin, including inflammatory bowel disease (IBD). In this study, the utility of serum S100A12, in discriminating IBD from irritable bowel syndrome (IBS), was tested.

**Methods:**

S100A12 serum levels were determined in 64 patients with ulcerative colitis (UC), 64 with Crohn's disease (CD) and 73 with IBS, by means of an enzyme-linked immunosorbent assay. S100A12 serum levels were evaluated with respect to the levels of known inflammatory markers and patients' characteristics.

**Results:**

The median values of serum S100A12 levels were 68.2 ng/mL (range: 43.4-147.4) in UC, 70 ng/mL (41.4-169.8) in CD and 43.4 ng/mL (34.4-74.4) in IBS patients. UC and CD patients had significantly higher serum S100A12 levels compared to IBS patients (*P *= 0.001 for both comparisons). Moreover, a cut-off for serum S100A12 levels of 54.4 ng/mL could predict both UC and CD with a 66.7% sensitivity and a 64.4% specificity. The area under curve was estimated at 0.67 with a 95% confidence interval of 0.60-0.75 (*P *< 0.001). Considering standard activity indices, higher serum S100A12 levels in active compared to inactive IBD were observed, although the recorded difference did not reach statistical significance. C-reactive protein (CRP) and serum amyloid A (SAA) levels, showed a statistically significant positive correlation with S100A12 (r = 0.39, *P *= 0.001 and r = 0.23, *P *= 0.02 respectively).

**Conclusions:**

Increased levels of circulating S100A12 are found in IBD, compared to IBS. When used to distinguish IBD from IBS adult patients, serum S100A12 levels exhibit moderate performance. On the other hand, serum S100A12 may serve as an inflammatory marker in IBD, since it is well correlated with CRP and SAA.

## Background

Inflammatory bowel disease (IBD) remains a diagnostic challenge for the clinician who faces a repertoire of diverse and fluctuating symptoms and signs. It is mainly due to a subtle or atypical presentation that in some cases the discrimination of Crohn's disease (CD) or ulcerative colitis (UC), from other diseases of the alimentary tract, especially from irritable bowel syndrome (IBS), becomes rather problematic [1]. On the other hand, although several markers are used for diagnostic purposes [2] a simple, economic and reliable test, as an alternative to more complex procedures for IBD diagnosis, is still under investigation. It is in this eager environment that S100A12 has emerged as a promising marker for both diagnosis and disease activity in IBD [3,4]. The S100A12, also known as calgranulin C, is a member of the S100 protein family which, in humans, consists of twenty five EF-hand (*a *helix-loop-*a *helix), calcium-binding proteins, of which the vast majority is in a homodimer, heterodimer ie S100A8/A9, or more complex form [5]. S100A12, like S100A8/A9 (calprotectin), is considered phagocyte-specific, exhibits proinflammatory properties and has already been linked to many different diseases of inflammatory origin, including IBD [3,4]. Several studies using the determination of S100A12 in feces, revealed a significant association between fecal S100A12 levels and IBD,[6-8] and especially active disease [8], an association that could also aid differential diagnosis by distinguishing IBD from IBS [9]. A limited number of studies was also conducted to examine the diagnostic potential of the S100A12 levels in serum. To our knowledge, three studies have been published so far, two carried out in pediatric [7,10] and one in adult populations [8]. The present study, however, is the first testing the utility of serum S100A12 levels in discriminating IBD from IBS in adults, while at the same time examining serum S100A12 titers with respect to disease type (UC or CD) or activity, traditional markers of disease activity, patients' characteristics and treatment modalities.

## Methods

### Patients

A total of 201 patients followed at the Gastroenterology Department of the University Hospital of Larissa, Greece were recruited. Among these, 64 were UC and 64 CD patients. Mean age ± SD was 51.2 ± 13.4 years for the UC and 40.3 ± 15.6 years for the CD group. The control group consisted of 73 outpatients, with mean age of 46.7 ± 12.3 years, presenting with diarrhea ± abdominal pain, who were diagnosed, after a full work-up (endoscopies, histopathology, cultures etc), and classified as D-IBS or M-IBS (51 and 22 individuals, respectively) according to Rome III criteria [11]. The control group was both age (*P *= 0.692) and sex (*P *= 0.126) matched with IBD patients. The demographic and clinical characteristics of UC and CD patients are presented in Table 1.

**Table 1:** Demographic and clinical characteristics for UC and CD group

	UC		CD	
	
	N	%	N	%
**Total**	64		64	

**Sex**				
Men	40	62.5	35	54.7
Women	24	37.5	29	45.3
**Age, mean (SD)**	51.2(13.4)		40.3(15.6)	
**Age at onset**				
≥40 yrs	-		11	17.2
<40 yrs	-		53	82.8
**Current smoking**				
No	54	84.4	21	32.8
Yes	10	15.6	43	67.2
**Disease duration**, **mean (SD), years**	7.1(0.3)		5.3(0.3)	
**Disease extent (UC)**				
Proctitis	18	28.1	-	-
Left-sided colitis	22	34.4	-	-
Pancolitis	24	37.5	-	-
**Disease location (CD)**				
Ileitis	-	-	22	34.4
Colitis	-	-	11	17.2
Ileocolitis	-	-	31	48.4
**Disease behavior**				
Stricturing	-	-	14	21.9
Penetrating	-	-	9	14.1
NS/NP	-	-	41	64
**Extraintestinal manifestations**				
None	43	67.2	34	53.1
1 or more	21	32.8	30	46.9
**Treatment**				
5-ASA	49	76.6	54	84.4
Corticosteroids	25	39.1	46	71.9
Immunosuppressants	14	21.9	21	32.8
Anti-TNF	3	4.7	16	25.0
Surgery	1	1.6	2	3.1

UC: ulcerative colitis, CD: Crohn's disease, yrs: years, NS/NP: non stricturing non penetrating

The diagnosis of IBD was established upon the co-evaluation of findings originating from clinical and endoscopic procedures, imaging studies, histopathology and laboratory analyses. Disease activity in the IBD group was documented using conventional indices: Crohn's Disease Activity Index (CDAI) [12] and the Clinical Activity Index (CAI), for UC [13]. A CDAI score greater than 150 and a CAI score exceeding 4, on a 0-16 scale, were considered as active CD and active UC, respectively. Disease location and behavior, in CD, were determined using the Vienna classification [14] whereas for disease extent, in UC, the Montreal classification [15] was used.

### Sample Collection and Preparation

Blood samples were collected in serum separator tubes and were allowed to clot for 30 minutes. All samples were then centrifuged and the obtained serum was stored at -25°C, for later analysis. The pre-analytical phase, including sampling and handling methods (sampling tubes, storage conditions etc.) was identical in all cases.

### Laboratory Assays

#### S100A12 Assay

Serum S100A12 levels were determined by means of a sandwich enzyme-linked immunosorbent assay (ELISA), using a human diagnostic kit (INFLAMARK) produced by CIS bio international (France). All samples were measured in duplicate (50 μl) within a standard range of 0-3000 ng/ml and with a total incubation time of 2 hours and 10 minutes, in room temperature, using two monoclonal antibodies, one in the solid phase (coated micro plates) and the other in the conjugate buffer. The detection limit of the assay was set at 20 ng/ml. If the difference between duplicate results of a sample was more than 5%, the sample assay was repeated, while the in-run coefficient of variation was 4.1%.

#### CRP and SAA Assays

The determination of CRP and SAA was performed by means of immunonephelometry with the Behring Nephelometer Analyzer II (BNII). For CRP and SAA measurements, the N High Sensitivity and the N Latex SAA kits (Dade Behring Gmbh, Germany) were used, respectively. The control and standard sera were provided by the same company, and used according to the manufacturer's instructions.

### Statistical Analysis

Variables were first tested for normality. Normal variables are expressed as mean ± SD (standard deviation) while variables with skewed distribution are expressed as median (interquantile range). If the normality assumption was satisfied for the comparison of means between two groups, Student's t-tests were used and Mann-Whitney tests otherwise. For multiple group comparisons the Kruskal-Wallis test was applied. Qualitative variables are expressed as absolute and relative frequencies. S100A12 was tested for its ability to predict UC and CD using receiver operating characteristic (ROC) curves. The overall performance of the ROC analysis was quantified by computing area under the curve (AUC). An area of 1 indicated perfect performance, while 0.5 indicated a performance that was not different than chance. Using ROC analysis the cut-off values, with the optimal sensitivity and specificity, for the prediction of UC and CD, were also calculated. Spearman correlation coefficients were used to explore the association of S100A12 with SAA and CRP values, CDAI and CAI, as well as disease duration. *P*-values for S100A12 with respect to the severity of disease were also computed. For the comparison of proportions, chi-square tests were used. All *P *values are two-tailed. Statistical significance was set at *P *< 0.05 and analyses were conducted using STATA statistical software (version 6.0).

### Ethical Considerations

Informed consent was obtained from all patients included in the present study, along with a verbal permission for the use of the samples acquired for scientific research. The study was approved by the ethical committee of the School of Medicine of the University of Thessaly, in Larissa, Greece.

## Results

### S100A12 levels with regard to disease characteristics

The median value of serum S100A12 levels was 68.2 ng/mL (range: 43.4-147.4) for UC patients and 70 ng/mL for CD patients (range: 41.4-169.8). IBS patients, on the other hand, exhibited a median of 43.4 ng/mL (range: 34.4-74.4) while no statistically significant differences were observed between D-IBS (median: 45.2 ng/mL, range: 34-85.1) and M-IBS (median: 44.1 ng/mL, range: 36.7-87.5) patients (*P *= 0.89). The recorded difference in S100A12 median values between UC and IBS was significant (*P *= 0.001) and this was also the case when CD and IBS median values were compared (*P *= 0.001).

**Figure 1:**
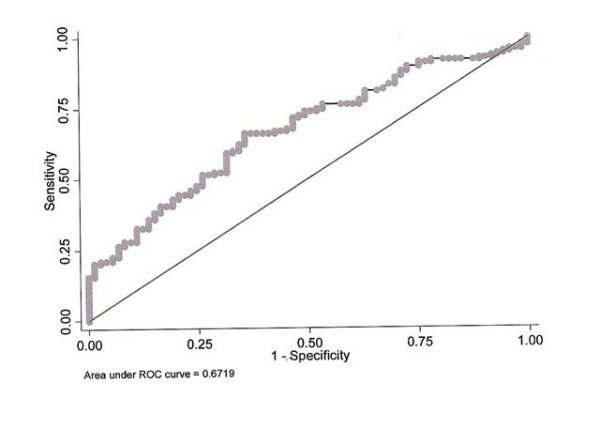
**ROC curve for the prediction of both ulcerative colitis and Crohn's disease from serum S100A12**.

ROC curve analysis showed that the optimal cut-off of S100A12 for the prediction of UC was 54.4 ng/ml, with a 68.8% sensitivity and a 64.4% specificity. Similarly, the S100A12 serum value of 55 ng/mL represented the optimal cut-off for the prediction of CD, with a sensitivity of 64.1% and a specificity of 64.4%. The area under the curves (AUCs) was 0.67 (95% CI: 0.58-0.76) and 0.67 (95% CI: 0.58-0.76), for the prediction of UC and CD, respectively, and statistically significant (*P *= 0.001, in both cases). Furthermore, ROC analysis (Figure 1) demonstrated that when a cut-off for S100A12 serum levels of 54.4 ng/mL was used, both UC and CD could be predicted with a 66.7% sensitivity and 64.4% specificity. The AUC, in this case was 0.67 (95% CI: 0.60-0.75) (*P *< 0.001).

Serum S100A12 levels were also studied while considering disease activity. S100A12 serum levels of IBD patients with active and inactive disease were both higher than those of IBS individuals (*P *= 0.02 in both cases). Although patients with active disease seemed to exhibit an increased median of 70.5 (42.6-142.2 ng/mL) compared to that of patients with inactive IBD, which was 64.5 (43.1-192.3 ng/mL), this difference did not prove significant (*P *= 0.546). Likewise, when the association of serum S100A12 with CDAI and CAI was examined no significant correlations emerged (Spearman r: 0.08 and 0.07, respectively and *P *≈ 0.4, in both cases).

A similar observation was made when S100A12 levels were compared among CD patients with diverse disease behavior: stricturing, penetrating and non-stricturing non-penetrating (ns/np). The performed analysis showed no associations between S100A12 and CD behavior, since patients with stricturing (median: 62.1 ng/mL, range: 32-141.2 ng/mL), penetrating (median: 73.5 ng/mL, range: 41.2-136.7 ng/mL) or ns/np (median: 78.4 ng/mL, range: 29.3-157.6 ng/mL) disease had comparable levels (*P *= 0.321).

The variations in the median of serum S100A12 did not show a significant association with either disease extent, in UC (*P *= 0.590) or disease location in CD (*P *= 0.512) (Table 2). Similar results were obtained when disease duration was taken into account. S100A12 serum concentration was not associated with disease duration since a low Spearman correlation coefficient (r: 0.09) and level of significance (*P *= 0.532) were recorded.

**Table 2:** Median values of S100A12 according to the extent of UC or location of CD

S100A12			
		
	N	Median (range)	P*
**UC**			
Proctitis	18	50.4(33-78.3)	0.590
Left-sided colitis	22	102.1(54.4-142.3)	
Pancolitis	24	62.6(43.4-141.2)	
**CD**			
Ileitis	22	122.8(47.8-199.5)	0.512
Colitis	11	34.3(29-40.1)	
Ileocolitis	31	70.5(49.1-289.8)	

UC: ulcerative colitis, CD: Crohn's disease

*Kruskal-Wallis test

Serum S100A12 levels were also examined with respect to the presence of one or more IBD-related extraintestinal manifestations. No significant differences regarding S100A12 values were found betwen IBD subjects with (median: 63.9 ng/mL, range: 38.6-153.7 ng/mL) or without (median: 78.5 ng/mL, range: 51.6-156.9 ng/mL) extraintestinal manifestations (*P *= 0.293).

### S100A12 levels with focus on patients' characteristics

S100A12 did not fluctuate significantly when male (median: 69.5 ng/mL, range: 43.1-167.3 ng/mL) and female (median: 70.5 ng/mL, range: 42.6-123.8 ng/mL) IBD patients were compared (*P *= 0.729). Similarly, no statistically significant differences or trends were recorded when serum S100A12 levels were studied under the light of age at onset (*P *> 0.1).

Considering the smoking habits of subjects in the IBD group, although at first glance, higher S100A12 serum levels in UC non-smokers (median: 72.4 ng/mL, range: 47.6-152.6 ng/mL) compared to UC current smokers were observed, this difference did not reach statistical significance (*P *= 0.215). Similarly, although higher S100A12 serum levels in CD patients were related to a current smoking status (median: 78.0 ng/mL, range: 40.1-156.9 ng/mL), the difference between CD current smokers and CD non-smokers was also non-significant (*P *= 0.592) (Table 3).

**Table 3:** Median values of S100A12 according to smoking status

					S100A12					
			IBD			UC			CD	
		N	Median (range)	P*	N	Median (range)	P*	N	Median (range)	P*
Smoking	No	75	64.5 (44.2-154)	0.813	54	72.4 (47.6-152.6)	0.215	21	56 (42.6-199.5)	0.592
	Yes	53	78.0 (39.1-142.2)		10	54.6 (28.1-133.2)		43	78 (40.1-156.9)	

UC: ulcerative colitis, CD: Crohn's disease

*Mann-Whitney test

Finally, the levels of serum S100A12 did not seem to have any correlation with treatment modalities (5-ASA, corticosteroids, immunosuppressants, anti-TNF) (*P *> 0.05 in all cases) (Table 4). As for the need for surgery, safe comparison could not be performed since surgical interventions had been applied in only three cases.

**Table 4:** Median values of S100A12 according to clinical characteristics and treatment

			S100A12	
		N	Median (Interquartile range)	P*
Activity	Active	40	70.5(42.6-142.2)	0.546
	Inactive	88	64.5(43.1-192.3)	
Extraintestinal manifestations	None	77	63.9(38.6-153.7)	0.293
	≥1	51	78.5(51.6-156.9)	
5-ASA	No	25	61.2(34.3-133.2)	0.210
	Yes	103	74.4(44.2-157)	
Corticosteroids	No	57	63.9(37.3-136.4)	0.285
	Yes	71	74.4(47.6-157)	
Immunosuppressants	No	93	64.5(43.1-142.2)	0.340
	Yes	35	83.5(42.9-282.0)	
Anti-TNF	No	109	69.5(43.6-152.6)	0.896
	Yes	19	70.5(38.6-157.0)	

*Mann-Whitney or Kruskal-Wallis tests

### S100A12 with respect to CRP and SAA levels

S100A12 was also examined with respect to the known inflammatory markers CRP and SAA. Patients with active IBD had statistically significant higher CRP levels (median: 2.27 mg/dL, range: 0.75-31.15 mg/dL) compared to patients with inactive disease (median: 1.10 mg/dL, range: 0.00-19.20 mg/dL), (*P *= 0.002). Similarly, SAA levels were greater in active (median: 8.66 mg/dL, range: 0.20-84.4 mg/dL) than in inactive IBD (median: 6.25 mg/dL, range: 0.10-58.8 mg/dL), (*P *= 0.001). A significant positive correlation was found between SAA and S100A12 (r = 0.22, *P *= 0.018) as well as between CRP and S100A12 (r = 0.35, *P *< 0.001).

## Discussion and Conclusions

IBD has been long ago recognized as a systemic inflammatory entity and as such, it is anticipated to induce changes exceeding the boundaries of bowel mucosa, being reflected in a broader spectrum of tissues, including blood [16-19]. Examples of such changes are the fluctuations in the levels of CRP, SAA, TNF-α, Interleukins [16], S100 proteins [17], metalloproteinases [18], angiogenins [19] etc. Some of these substances have been introduced as inflammatory markers and used in clinical practice for diagnostic purposes in patients with suspected or confirmed IBD. The sufficiency of these markers, however, has been challenged repeatedly due to a moderate performance, making the study of new markers in IBD mandatory [17,20,21]. There are numerous studies examining the significance of several fecal, serum or mucosal markers including members of the S100 protein family such as S100A8/9 and S100A12 [3,4,8,22,23]. Since the vast majority of available literature is focused on S100A8/9 [22,23] and to a lesser extent on S100A12 [6-10] and their role in IBD, a subsequent gap in the literature regarding S100A12 studies in IBD patients originated. Moreover, two serum S100A12 studies were carried out in children [7,10] while only one was conducted in an adult population [8].

Over the past years, several markers emerged to facilitate the differentiation of IBD from IBS. The most promising among these were fecal S100A8/A9 [24-26] and more recently fecal S100A12, which has been shown to discriminate IBD from IBS with a sensitivity of 86% and specificity of 96% [9]. In our study, a significant elevation in serum S100A12 levels correlated well with IBD but not with IBS, thus allowing the distinction between the two entities. The performed analysis also focused on the determination of a cut-off for IBD prediction that would exhibit the highest possible sensitivity and specificity. This optimal cut-off was estimated at 54 ng/mL and was shown to predict both CD and UC with a sensitivity of 66.7% and a specificity of 64.4%, in the population of the study. Although, this performance is superior to the one reported by Sidler et al (21% sensitivity and 81% specificity) [7] still remains moderate and evidently, lower than that reported for fecal S100A12 [9], probably due to the fact that fecal stream is in direct contact with the inflamed mucosa. Perhaps, a better diagnostic performance could be established while assessing simultaneously S100A12 and other IBD-related diagnostic markers, in serum, a model already applied when anti-glycan antibody determinations are carried out [27].

As shown in the results section, a significant correlation between S100A12 serum levels and disease activity or factors (ie treatment) that can alter the activity could not be established. Both UC and CD patients with active disease seemed to have higher values of S100A12 in serum, compared to IBD subjects with inactive disease but this difference was not statistically significant. On the other hand, this "flaw", could also be regarded as a beneficial characteristic, as it implies that the value of serum S100A12 for discrimination of IBD from IBS cannot be challenged by the presence of inactive disease. Although our findings could be the result of the rather small number of patients recruited in the study, they are in accordance with previous reports on the inadequacy of serum S100A12 as a marker for monitoring IBD activity [9], although in an earlier study, S100A12 concentration in serum has been shown to differentiate active from inactive IBD [8]. In that study, however, the levels of serum S100A12 in UC patients with inactive disease were comparable to those in healthy controls [8].

According to our results, serum S100A12 determination could not be used to predict disease extent in UC patients, since the S100A12 serum values were not significantly different in patients with proctitis, left-sided colitis or pancolitis. Similarly, by relying on S100A12 serum levels, disease location could not be predicted, despite the fact that, at first glance, patients with ileocolitis or ileitis seemed to exhibit higher values of serum S100A12, compared to those with CD colitis alone. The data presented by Foell et al, on the other hand, were indicative of a predominant mucosal release of S100A12, from the inflamed colon of CD patients. In the same study, S100A12-stained neutrophils which adhered to the endothelium of blood vessels were detected in the CD-affected terminal ileum [28]. Although no hard evidence favoring this assumption are available, it would be useful to examine the possibility that the S100A12 in serum of CD patients may derive mainly from neutrophils migrating through the blood vessels into the diseased ileum or that ileal inflammation triggers a more "systemic" type of response.

As for the study of S100A12 serum levels with respect to the presence of any extraintestinal manifestations, no significant variations became evident. S100A12 serum concentrations were comparable between IBD patients with or without extraintestinal manifestations.

The effect of current smoking status on serum S100A12 concentration, in IBD individuals was also examined, since smoking has been traditionally regarded as a factor which could improve UC and worsen CD [29]. As shown above, the levels of S100A12 in serum of current smokers, diagnosed with CD appeared to be elevated, compared to CD non-smokers. In the UC group, the levels of serum S100A12 seemed to be higher in non-smokers than in those found in current smokers. These findings, although within reason, lack credibility since, neither statistical significance nor trend could be reached for any of the recorded smoking-related associations.

Similarly, all treatment modalities, leading to a subsequent disease remission, did not seem to have a noticeable effect on serum levels of S100A12 in the IBD group. This result is partly in accordance with an earlier study in which corticosteroids did not alter significantly S100A12 serum levels of IBD adults [8]. The impact in S100A12 serum levels of infliximab-treated IBD subjects recorded in that study [8] was not verified in ours.

What appears to be of interest is that a statistically significant association was found between serum S100A12 levels and the well-known markers of inflammation, CRP and SAA, in the absence of a similar association with IBD activity-determined by conventional inflammatory indices. Before discarding serum S100A12 as a potential marker for the monitoring of IBD activity it would, perhaps be better if possible associations with other indices [30,31] were also evaluated.

At this point a potential study limitation has to be underlined. The serum S100A12 levels determined in our population, using the prototypic ELISA described above, were lower compared to IBD-oriented studies, using different ELISA assays [7,8,10]. According to the manufacturer (CIS bio international, France) this is probably the result of discrepancies, originating from between-studies differences in the pre-analytical phase, as well as the inability of the specific kit to perform equally well when different blood-containing mediums were used. In line with this notion are the results from a study on the impact of diverse sample handling conditions -incubation in tubes with gel/anticoagulants/calcium, dilution with calcium containing buffers, different storing conditions, repeated freeze-thaw cycles etc- revealing great fluctuations in the S100A12 levels, even in blood samples obtained from healthy individuals [32]. According to the authors, however, the most reliable blood sample for S100A12 determinations would be serum stored in gel-containing tubes, as in our study [32]. The use of the most suitable -or least unsuitable- type of blood-derived sample, along with the establishment of identical for all samples "handling" throughout the critical phase that preceded analysis, minimise the odds that the between-subjects differences recorded in our study are due to the use of the specific ELISA.

In conclusion, it is evident that the upregulation of S100A12 is not confined to the boundaries of an IBD-occupied intestine but is also reflected systemically and subsequently detected in serum. This increase in serum S100A12 is associated with IBD and is also well correlated with the "classic" markers of inflammation CRP and SAA. On the other hand, since the diagnostic utility of serum S100A12 was moderate, when used alone, its use in a "palette" of established serological markers, might actually lead to an improved overall diagnostic performance. Thus, it seems that further research in larger populations is mandatory, in order to verify these results, examine the association of serum S100A12 with disease or patients' characteristics and specify whether serum S100A12 could be added in the existing armamentarium, used for the diagnosis of IBD.

## Competing interests

The authors declare that they have no competing interests.

## Authors' contributions

ACM, ANK, PG, SPP, participated in study design, diagnostic procedures, interpretation of results and drafting of manuscript. AK and VV carried out laboratory analyses. CT performed statistical analysis. All authors read and approved final manuscript

## Pre-publication history

The pre-publication history for this paper can be accessed here:

http://www.biomedcentral.com/1471-230X/10/118/prepub
